# Bacteremia Caused by a Serotype Ob5 Vibrio cholerae Strain in a Cirrhotic Patient in China

**DOI:** 10.1128/spectrum.02054-23

**Published:** 2023-06-28

**Authors:** Xiaohong Xu, Jiao Qian, Qinjian Ke, Yizhang Wang, Yanchao Liu, Danni Bao

**Affiliations:** a Department of Clinical Laboratory, Sanmen People’s Hospital, Taizhou, Zhejiang, China; b Department of Clinical Laboratory, Taizhou Hospital of Zhejiang Province, Wenzhou Medical University, Taizhou, Zhejiang, China; c Key Laboratory of System Medicine and Precision Diagnosis and Treatment of Taizhou, Taizhou, Zhejiang, China; d Department of Laboratory Medicine, First Affiliated Hospital, Zhejiang University School of Medicine, Hangzhou, Zhejiang, China; e Key Laboratory of Clinical In Vitro Diagnostic Techniques of Zhejiang Province, Hangzhou, Zhejiang, China; University of Maryland Eastern Shore

**Keywords:** *Vibrio cholerae*, bacteremia, genome sequencing, liver cirrhosis, non-O1/non-O139

## Abstract

The increasing incidence of non-O1/non-O139 Vibrio cholerae (NOVC) has been observed worldwide. However, septicemia caused by NOVC remains a rare condition that has received limited attention. Currently, there are no established treatment guidelines for bloodstream infections caused by NOVC, and the understanding of this condition mainly relies on individual case reports. Although NOVC bacteremia can be fatal in a small percentage of cases, knowledge about its microbiological features remains limited. Here, we present a case of V. cholerae septicemia caused by NOVC in a 46-year-old man with chronic viral hepatitis and liver cirrhosis. The isolated strain, named V. cholerae VCH20210731 and classified as a new sequence type (ST), ST1553, was found to be susceptible to most of the antimicrobial agents tested. O-antigen serotyping of V. cholerae VCH20210731 revealed that it belonged to serotype Ob5. Interestingly, the *ctxAB* genes, which are typically associated with V. cholerae, were absent in VCH20210731. However, the strain possessed 25 other potential virulence genes, such as *hlyA*, *luxS*, *hap*, and *rtxA*. The resistome of V. cholerae VCH20210731 included several genes, including *qnrVC4*, *crp*, *almG*, and *parE*. Nevertheless, susceptibility testing demonstrated that the isolate was susceptible to most of the antimicrobial agents tested. Phylogenetic analysis indicated that the closest strain to VCH20210731 was strain 120 from Russia, differing by 630 single-nucleotide polymorphisms (SNPs). Our findings contribute to the understanding of the genomic epidemiological characteristics and antibiotic resistance mechanisms of this invasive bacterial pathogen.

**IMPORTANCE** This study highlights the discovery of a novel ST1553 V. cholerae strain in China, providing valuable insights into the genomic epidemiology and global transmission dynamics of V. cholerae. It is important to note that clinical presentations of NOVC bacteremia can vary significantly, and the isolates demonstrate genetic diversity. Consequently, health care professionals and public health experts should remain vigilant about the potential for infection with this pathogen, particularly considering the elevated prevalence of liver disease in China.

## OBSERVATION

The most commonly reported non-O1/non-O139 Vibrio cholerae (NOVC) infections include gastroenteritis, otitis, and soft tissue infections. This comma-shaped bacillus, which is found in aquatic environments, can infect humans through contaminated water or food (1). V. cholerae can be classified into more than 200 serotypes based on changes in its lipopolysaccharide surface O antigen (2). O1 and O139 strains have been associated with typical cholera epidemics throughout the world (2), while NOVC isolates do not produce the cholera-causing toxin. Although NOVC can cause diarrhea on occasion, it typically causes invasive extraintestinal disease and bacteremia in individuals with liver disease or immunocompromised status (3). To our knowledge, the first case of NOVC bacteremia was reported from the United States in 1974 (4). Few studies have focused on the clinical, epidemiological, and genetic characteristics of NOVC bacteremia in China. However, due to the various clinical manifestations of NOVC bacteremia and the genetic diversity among isolates, it is crucial for clinicians and public health experts to be more vigilant to prevent the possibility of infection, especially for Chinese individuals with a high prevalence of liver disease. Our study provides new insights into the genomic epidemiological characteristics and global transmission dynamics of V. cholerae.

After a blood culture tested positive, β-hemolytic, oxidase-positive colonies were observed after 24 h of culture on blood agar. Subsequently, colonies were subcultured on thiosulfate-citrate-bile salts-sucrose (TCBS) agar and appeared as large yellow colonies. Identification of V. cholerae was confirmed by matrix-assisted laser desorption ionization–time of flight mass spectrometry (MALDI-TOF MS) (Vitek MS; bioMérieux, France). NOVC was identified using polyvalent O1 and O139 antisera in slide agglutination tests. Vitek 2 Compact (bioMérieux) antimicrobial susceptibility testing was used to determine the susceptibility of V. cholerae VCH20210731 to most antimicrobial agents. Antimicrobial resistance testing was performed for V. cholerae VCH20210731 using the broth microdilution method with the following antimicrobial agents: amikacin, ampicillin, gentamicin, ceftazidime, ceftriaxone, imipenem, ciprofloxacin, levofloxacin, cefuroxime, cefazolin, and azithromycin. The specimen was initially inoculated overnight at 37°C on Columbia blood agar, and a single colony of the target strain was grown overnight at 37°C in Mueller-Hinton broth (Oxoid Ltd., Basingstoke, UK). The bacterial species was identified using MALDI-TOF MS (Vitek MS; bioMérieux) and verified using 16S rRNA gene sequencing. MICs of antimicrobial agents were interpreted using Clinical and Laboratory Standards Institute (CLSI) guidelines (5).

Draft genome sequencing of V. cholerae VCH20210731 was undertaken using short-read sequencing with the NovaSeq 6000 platform (Illumina Inc., San Diego, CA, USA) to investigate the mechanisms of antimicrobial resistance. The genome sequence was automatically annotated using the NCBI Prokaryotic Genome Annotation Pipeline (PGAP). *In silico* multilocus sequence typing (MLST) analysis was performed by using the PubMLST database (6), while O-antigen genes, cholera toxin (CTX) prophages, sulfamethoxazole-trimethoprim (SXT) elements, antimicrobial resistance genes (ARGs), *Vibrio* pathogenicity islands (VPIs) (VPI-1 and VPI-2), and *Vibrio* seventh pandemic islands (VSPs) (VSP-I and VSP-II) in the genome were predicted using VicPred (http://vicpred.hanyang.ac.kr) (7).

Additionally, we retrieved 770 human V. cholerae strains and their related clinical information from the NCBI genome database (https://www.ncbi.nlm.nih.gov/genome) and analyzed the V. cholerae serotypes using Pathogenwatch (https://pathogen.watch). The BacWGSTdb v2.0 database was utilized for MLST analysis of the strains (8). Finally, single-nucleotide polymorphism (SNP) identification of V. cholerae was performed and a phylogenetic tree was constructed using Snippy v4.6.0 (https://github.com/tseemann/snippy) and FastTree (9), respectively; the phylogenetic tree was visualized using the Interactive Tree Of Life (iTOL) v5 web server (10).

This report describes the detection of V. cholerae VCH20210731 from a blood sample obtained in July 2021 from a 46-year-old man who was hospitalized with chronic viral hepatitis and liver cirrhosis. The patient had been experiencing fatigue and stomach distension for 3 days and had a history of hepatitis B cirrhosis for more than 5 years. On admission, his temperature was 36.9°C, heart rate 86 beats/min, and blood pressure 100/64 mm Hg. Physical examination revealed icteric sclera, minor ascites, splenomegaly, tremor in both upper limbs, spider nevus, and liver palms. Laboratory examination results were as follows: alanine aminotransferase activity, 17 U/L; aspartate aminotransferase activity, 23 U/L; total bilirubin level, 33.5 μmol/L; direct bilirubin level, 12.3 μmol/L; total protein level, 54.2 g/L; albumin level, 24.5 g/L; prothrombin time, 20.90 s; D dimer level, 8.44 mg/L; fibrinogen level, 1.02 g/L; white blood cell count, 1.50 × 10^9^ cells/L; hemoglobin level, 48.0 g/L; platelet count, 34 × 10^9^ cells/L. Cirrhosis with splenomegaly, gallbladder wall edema, splenic vein wall thrombosis, and abdominal fluid buildup were found on ultrasonography. After admission, the patient received treatment to improve blood circulation with infusion of 2 U red bloods cells. On July 15, the patient reported fever with chills, and his body temperature increased to 38.1°C.

NOVC infections in humans are usually associated with exposure to contaminated water or seafood (11). A large majority of patients report no such exposure, however, implying other plausible routes of transmission (1). Liver cirrhosis was more prevalent among patients with primary bacteremia than among those with other types of infections (3), and such patients should be closely monitored. This finding may due to various factors, such as high intestinal mucosal permeability caused by inflammation and edema, portal hypertension resulting in bypassing of the hepatic reticuloendothelial system, complement deficiencies, impaired phagocytosis, alterations in iron metabolism, and/or inefficient chemotaxis (12). It has also been hypothesized that liver disease and hematological malignancies frequently result in low platelet counts or poor coagulation function, which could facilitate bacterial entry into the systemic circulation and cause bacteremia. Additionally, specific strains of NOVC that generate hemolysin may be involved in bacterial invasion by promoting cell vacuolation and hemolysis (13).

In immunocompetent patients, NOVC gastroenteritis is usually self-limited and does not require the use of antibiotics. For complicated infections and immunocompromised patients, however, antimicrobial therapy is recommended (1). Dual-agent therapy for NOVC bacteremia has been proposed by several researchers (14). Combining a third-generation cephalosporin with a tetracycline or a fluoroquinolone appears to be effective, depending on susceptibility data. Treatment duration for septicemia is also debatable, ranging from 3 to 75 days in various publications (median, 14 days) (1).

Our patient displayed an atypical clinical manifestation of NOVC bacteremia, with only abnormal liver function test results, which were determined by ultrasonography to be due to liver cirrhosis, and only mild digestive symptoms, with diagnosis using blood culture. In addition, our patient achieved remission after only 2 days of treatment with ceftriaxone. The clinical manifestations of NOVC bacteremia have further proved to be diverse.

The draft genome sequence of V. cholerae VCH20210731 consists of 95 contigs, totaling 4,032,143 bp, and could be classified as a new sequence type (ST), ST1553. The overall G+C content of the strain was 47.6%, and a total of 3,623 coding sequences (CDSs) and 113 RNA genes (93 tRNA, 16 rRNA, and 4 noncoding RNA genes) were identified. O-antigen serotyping revealed that V. cholerae VCH20210731 belonged to serotype Ob5. The *ctxAB* genes are absent in V. cholerae VCH20210731, but the strain has 25 other putative virulence genes, such as *hlyA*, *luxS*, *hap*, and *rtxA*. Comparative genomic analysis also revealed that the isolate was nontoxigenic and possessed the CTX-RS1^−^ VPI^+^ VSP^+^ genotype. The resistome of V. cholerae VCH20210731 included several genes, such as *qnrVC4*, *crp*, *almG*, and *parE*. However, susceptibility testing showed that the isolate was susceptible to most of the antimicrobial agents tested, including amikacin, ampicillin, gentamicin, ceftazidime, ceftriaxone, ciprofloxacin, levofloxacin, cefuroxime, cefazolin, amoxicillin-clavulanic acid, piperacillin-tazobactam, trimethoprim-sulfamethoxazole, cefoperazone-sulbactam, and azithromycin (Table 1). Interestingly, the strain showed intermediate susceptibility to imipenem, with a MIC of 2 mg/L. However, the underlying reason for the occurrence of this phenotype remains unclear at present.

**TABLE 1:** Genotype and phenotypic resistance profile of strain VCH20210731

Antimicrobial agent	MIC (mg/L)		Susceptibility^*a*^	ARGs^*b*^
	Vitek 2	Broth dilution method		
Aminoglycosides				
Amikacin		4	S	
Gentamicin		1	S	
β-Lactams				
Ampicillin		4	S	
Ceftazidime	≤0.1	0.5	S	
Cefepime	≤0.1		S	
Cefazolin		2	S	
Cefuroxime	≤0.1	0.03	S	
Cefoxitin	≤4	2	S	
Amoxicillin-clavulanic acid	8		S	
Piperacillin-tazobactam	≤4		S	
Cefoperazone-sulbactam	≤8		S	
Imipenem	2	2	I	
Fluoroquinolones				*qnrVC4*, *parE*, *crp*
Ciprofloxacin		0.125	S	
Levofloxacin	≤1	0.25	S	
Macrolides				
Azithromycin		0.5	S	
Folate pathway inhibitors				
Trimethoprim-sulfamethoxazole	≤1/19	≤0.125/2.375	S	

a S, susceptible; I, intermediate.

b CRP, C-reactive protein.

**FIG 1:**
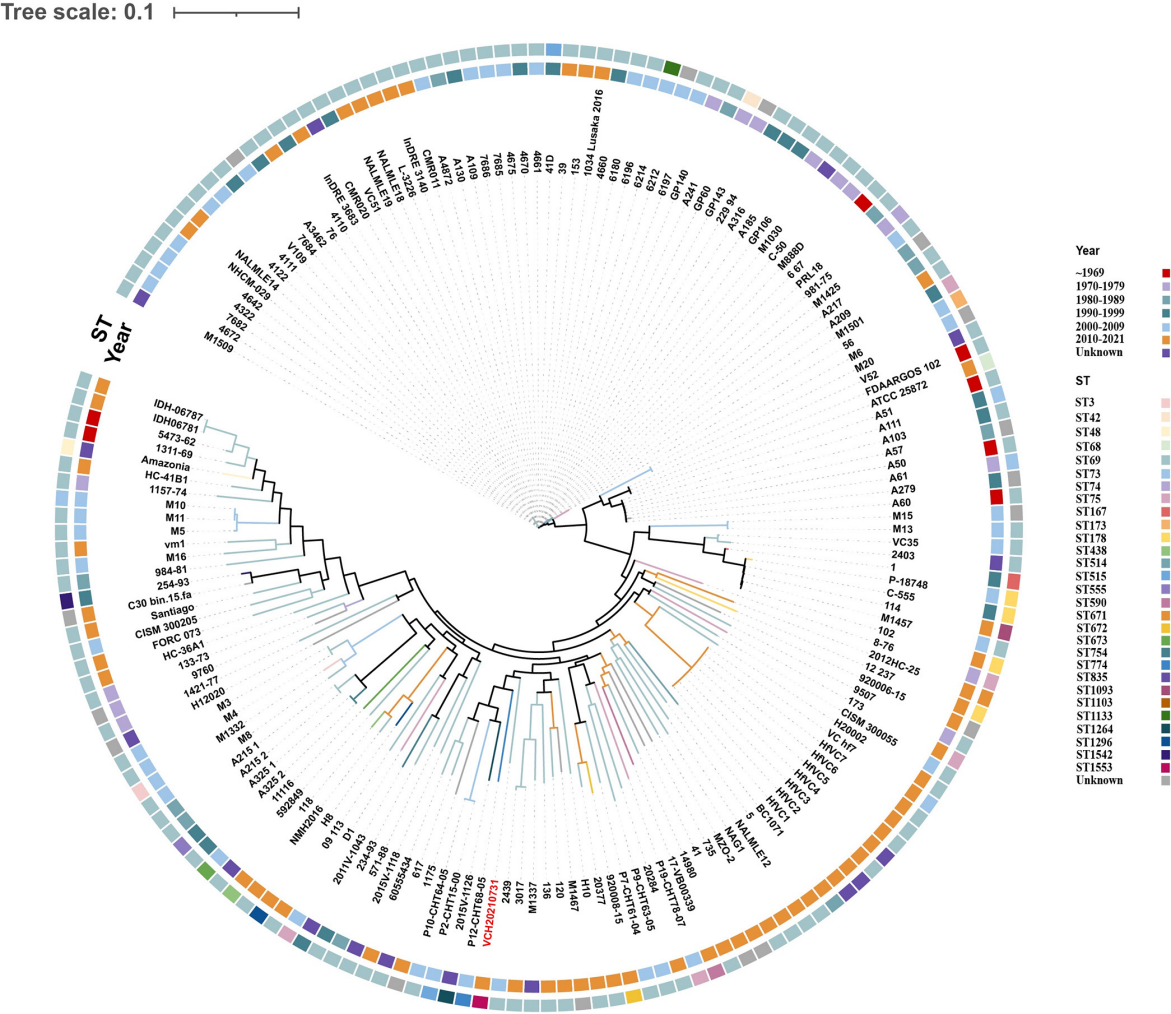
Recombination-filtered core genome phylogeny for 170 human V. cholerae isolates worldwide. The STs and isolation times of the strains are represented by squares of different colors. The strain isolated by us is highlighted in red.

**FIG 2:**
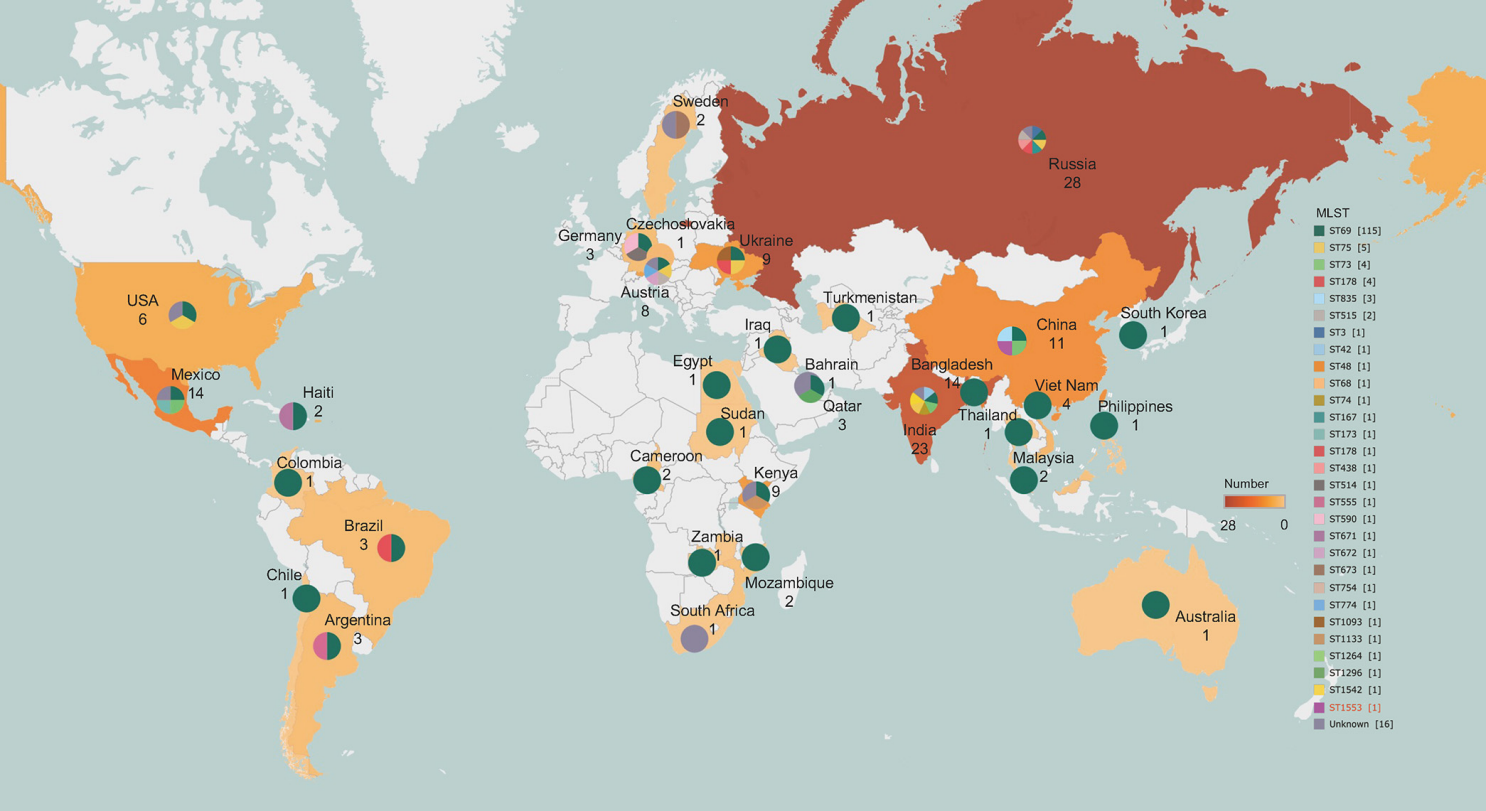
Geographical distribution of 170 NOVC strains worldwide. The presence and number of strains in different geographical locations are indicated by the shades of color. The pie charts show the frequency of distribution of different STs in each country (colors distinguish different STs). The numbers of strains for each ST are indicated in square brackets. The map was created with R software using the package ggplot2.

A total of 770 V. cholerae strains were isolated from 1949 to the present, including 574 O1 V. cholerae strains, 22 O139 V. cholerae strains, and 170 NOVC strains, with 2 anomalous assembly strains (which were removed) and 3 Vibrio paracholerae strains. The phylogenetic connections between V. cholerae VCH20210731 and the total of 170 NOVC strains currently deposited in the NCBI GenBank database were examined to evaluate the genomic epidemiological features of V. cholerae strains in a global context (Fig. 1). These strains are mainly distributed in Russia (28 strains [16.4%]), India (23 strains [13.5%]), Bangladesh (14 strains [8.2%]), Mexico (13 strains [7.6%]), and China (11 strains [6.4%]). They were found to have a variety of STs, with ST69 (114 strains [67%]), ST75 (5 strains [2.9%]), ST78 (4 strains [2.3%]), and ST178 (4 strains [2.3%]) being the most prevalent (Fig. 2). Moreover, V. cholerae VCH20210731 presents a distinctive ST, which we have submitted to the PubMLST database and designated ST1553. Unfortunately, the current availability of V. cholerae genomes in the NCBI database is limited, posing a challenge to conducting comprehensive analyses on the source tracking of this isolate. Phylogenetic analysis showed that the closest strain to our isolated strain VCH20210731 was strain 120 from Russia (NCBI assembly accession number GCF_008269365.1), with 630 SNPs.

In conclusion, we report a ST1553 V. cholerae strain from China that produced NOVC bacteremia. The global increase in sea surface temperatures poses a potential threat for the proliferation of pathogenic vibrios in aquatic environments, highlighting the need for awareness and vigilance. Our findings contribute to the understanding of the genomic epidemiological traits and antibiotic resistance mechanisms of this bacterial pathogen.

### Ethical approval.

This study was conducted in accordance with the Declaration of Helsinki, and approval was obtained from the Medical Ethics Committee at the Sanmen People’s Hospital, China. Written informed consent was provided by the patient to allow the case details to be published.

### Data availability.

The genome sequences of the chromosome of V. cholerae VCH20210731 have been deposited in NCBI GenBank under accession number JAJQMP000000000.
